# Resolving an 87-year-old taxonomical curiosity with the description of *Psylla frodobagginsi* sp. nov. (Hemiptera: Sternorrhyncha: Psyllidae), a second distinct *Psylla* species on the New Zealand endemic plant kōwhai

**DOI:** 10.1371/journal.pone.0221316

**Published:** 2019-09-18

**Authors:** Francesco Martoni, Karen Armstrong

**Affiliations:** Bio-Protection Research Centre, Lincoln University, Lincoln, New Zealand; Sichuan University, CHINA

## Abstract

A recent DNA-based assessment of the psyllid fauna of New Zealand recorded high genetic variation between populations that were expected to belong to the same psyllid species. Among these, a number of populations of the kōwhai psyllid *Psylla apicalis* (Ferris & Klyver, 1932), from a kōwhai species, *Sophora microphylla* Aiton (Fabaceae), presented high genetic variability. This gave new endorsement of an 87-year-old observation made by the entomologists Ferris and Klyver who, when describing the kōwhai psyllid, from *Sophora tetraptera* J.S. Muell., suggested that morphological variations could support more than one species. Accordingly, the morphological assessment conducted here, together with the genetic information now available, resulted in the description of *Psylla frodobagginsi* sp. nov. as a second New Zealand endemic psyllid species hosted by *S*. *microphylla*.

## Introduction

The superfamily Psylloidea (Hemiptera, Sternorrhyncha) comprises almost 4000 described species worldwide [1]. Yet, there are several indications that many more species remain undescribed (e.g. [2, 3]) which confounds a clear appreciation of the number of psyllid species, our ability to distinguish them and their distribution worldwide. Thus, there is a fundamental need to better understand their systematics and to confirm the most recent taxonomic classification [4] for the downstream applied benefits of that, such as studies on the insect/bacteria interactions and on their dispersal models (e.g. [5]). This can be facilitated by the continuous updating of psyllid taxonomic diversity and distribution. Good examples tackling Australasia and the Pacific, as a hot spot for biodiversity of the Psylloidea [1, 2], include the most recent works focusing on Australia [6, 7], New Zealand [3, 8, 9], Cook Islands [10] and Hawaii [11, 12]. Expediting this appraisal has been inclusion of molecular phylogenetic studies to enable a better understanding of this superfamily’s evolutionary history (e.g. [13, 14]. Equally this relies on a pre-existing and accurate understanding of psyllid diversity to ensure representative sampling across families and genera.

Within Australasia, the psyllid fauna of New Zealand can contribute useful information towards a global understanding of these insects [3, 8], with a less challenging number of species per geographic area to consider than Australia [1, 2]. Thus far, New Zealand is known to be home to 73 described psyllid species [8, 15, 16] and at least another 47 undescribed or hypothesised taxa [3, 17]; this compares with more than 500 species described to date in Australia [1, 2] and an unknown number of undescribed taxa present there [2]. Among these, the genus *Psylla*, comprised of 98 species worldwide and none in Australia [1], is to date represented in New Zealand by two described species, *P*. *apicalis* and *P*. *carmichaeliae* Tuthill, 1952, and a proposed additional five undescribed taxa [3].

The hypothesis of cryptic variation within taxa of the genus ‘*Psylla’* in New Zealand dated well before the advent of molecular analysis, which was the key driver for developing the current new description [3]. Essentially, when ‘*Psyllia* (sic) *apicalis’* was described as the first and only *Sophora*-feeding psyllid, the authors highlighted how this species showed such a variable morphology that it could as well be due to the presence of two separate taxa [18]. Of note was the different wing patterns and colours. Nonetheless, the authors assumed the presence of intergradation between the patterns and believed that the differences were merely variants of the same species [18].

More recently, molecular characters developed for the New Zealand Psylloidea, based on the cytochrome oxidase I (COI) barcode gene region [19], reported the presence of high genetic variability of 7–8% divergence between different populations of *P*. *apicalis*. This led to the hypothesis of the presence of up to five additional taxa belonging within this genus [3]. Since it is not the aim of this work to review the New Zealand psyllid species attributed to the genus *Psylla* per se, these additional taxa have been left in this genus. Rather, while awaiting a taxonomic review of *Psylla* to include a phylogenetic comparison of the New Zealand species and those elsewhere, this study focuses on an observation in the South Island that there was potentially more than one *Psylla* species associated with *S*. *microphylla*. In order to provide a useful and valid tool to distinguish specimens of a new species here as *Psylla frodobagginsi* and not *P*. *apicalis*, specimens of both species have been collected and a new morphological description for *P*. *frodobagginsi* plus re-description of *P*. *apicalis* is presented here which fully reflects the molecular divergence between them [3]. Measurements, figures and description are reported for both species, together with the information obtained from examination of the holotype of *P*. *apicalis*. This aims to contribute to a better understanding of the number of New Zealand endemic species belonging to this genus. In turn this will facilitate future work to reconsider more broadly the taxonomy, systematics and phylogenetics of the genus *Psylla*.

## Materials and methods

**Fig 1:**
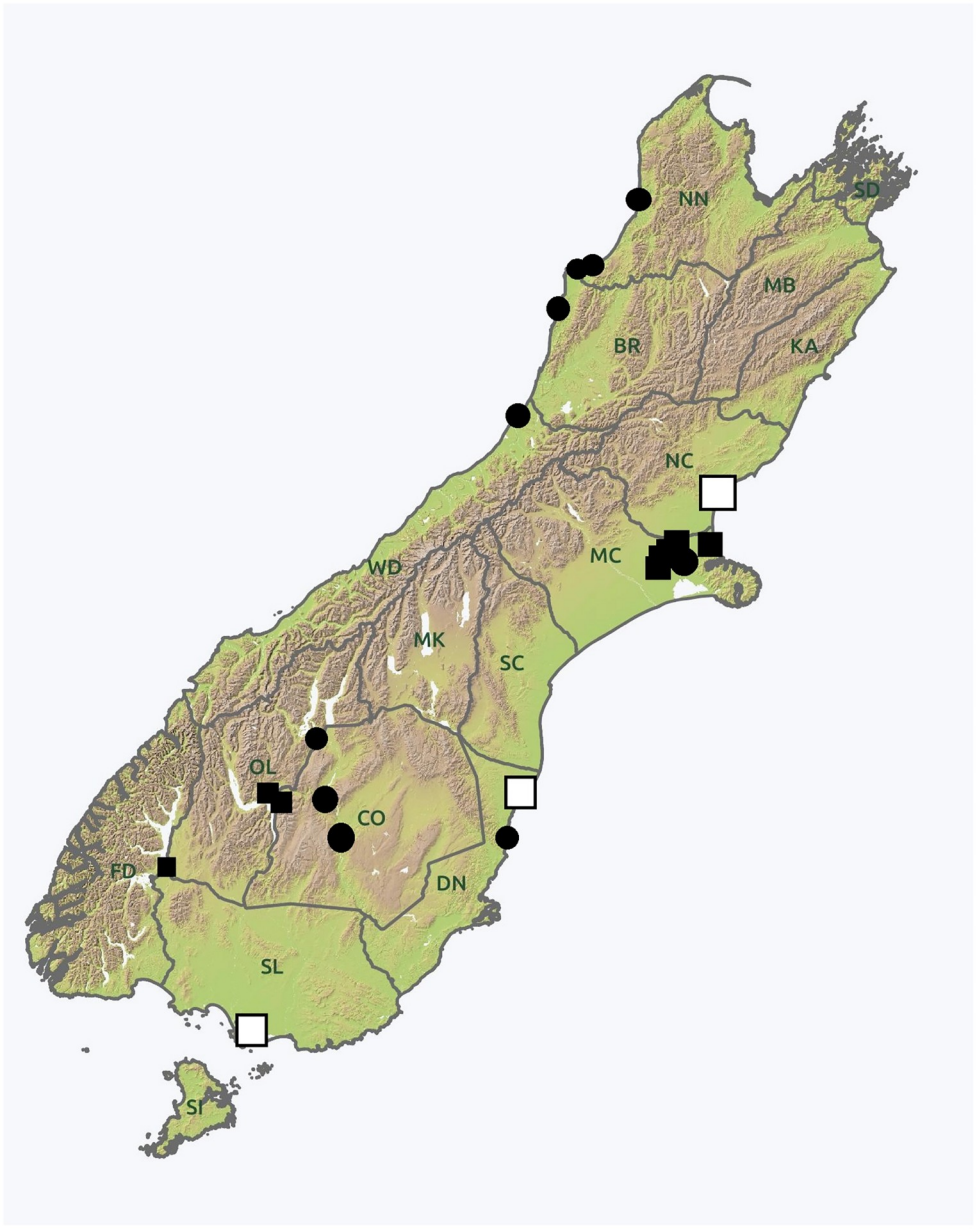
Map of the South Island of New Zealand, showing the 21 locations from which psyllids could be collected. Some of the locations included only *P*. *apicalis* (black squares), only *P*. *frodobagginsi* (black circles) or both the species (white squares). Regions delimitation follows Crosby et al. [20]. Reprinted from https://geographx.co.nz/ under a CC BY license, with permission from Geographx.

Psyllids were collected from kōwhai trees in 21 locations across 10 regions in the South Island of New Zealand, following Crosby’s regional delimitations [20] (Fig 1, Table 1). Collections were performed from public areas not requiring any permit. Samples were obtained by beating plant branches on a tray, then retrieving fallen insects using an entomological aspirator. Collected samples were stored, per tree, in high grade ethanol at -20°C.

**Table 1:** Populations of psyllids analysed in this study. The table provides information on the species, the population ID number, the collection date, the collectors, the GPS coordinates, the region within the South Island of New Zealand (based on Crosby’s regional boundaries [20]), the town location and the accession numbers for the DNA sequences previously available on GenBank for each population.

Species	ID	Date	Collector	Coordinate	Region	Location	COI	18S
*P*. *apicalis*	7	07-Sep-14	Martoni, F	43°38'23.3"S 172°28'30.8"E	MC	Lincoln	MG132469-70	MG195385
	187	01-Sep-15	Brown, SDJ	43°31'22.44"S 172°35'7.44"E	MC	Christchurch	MG132465	
	200	02-Nov-15	Martoni, F & Evans, H	45°5'13.47"S 170°58'31.85"E	DN	Oamaru		
	201	08-Nov-15	Martoni, F & Evans, H	46°24'09.7"S 168°21'38.2"E	SL	Invercargill	MG132466	
	204	08-Nov-15	Martoni, F & Evans, H	45°25'25.3"S 167°43'05.4"E	FD	Te Anau	MG132467	
	206	09-Nov-15	Martoni, F & Evans, H	45°02'12.8"S 168°39'38.3"E	OL	Queenstown	MG132468	
	208	09-Nov-15	Martoni, F & Evans, H	45°02‴S 168°39‴E	OL	Queenstown		
	429	10-Sep-16	Martoni, F	43°09'23.4"S 172°43'50.5"E	NC	Amberley		
	431	13-Oct-16	Martoni, F	43°38'31.4"S 172°28'15.1"E	MC	Lincoln		
	433	13-Oct-16	Martoni, F	43°38'25.0"S 172°28'28.9"E	MC	Lincoln		
	434	01-Oct-16	Bowie, M	43°38'14.0"S 172°29'09.0"E	MC	Lincoln		
*P*. *frodobagginsi*	55	30-Nov-14	Martoni, F	43°38‴S 172°28‴E	MC	Lincoln	MG132481	MG195386
	200	02-Nov-15	Martoni, F & Evans, H	45°5'13.47"S 170°58'31.85"E	DN	Oamaru	MG132471	
	201	08-Nov-15	Martoni, F & Evans, H	46°24'09.7"S 168°21'38.2"E	SL	Invercargill		
	209	09-Nov-15	Martoni, F & Evans, H	45°02'09.7"S 169°11'33.8"E	CO	Cromwell	MG132472	
	215	09-Nov-15	Martoni, F & Evans, H	45°15'06.3"S 169°23'19.3"E	CO	Alexandra	MG132473	
	218	10-Nov-15	Martoni, F & Evans, H	44°41'37.9"S 169°08'08.5"E	OL	Wanaka	MG132474	
	226	11-Nov-15	Martoni, F & Evans, H	42°42'34.4"S 170°58'25.8"E	WD	Hokitika	MG132475	
	230	11-Nov-15	Martoni, F & Evans, H	42°27'25.8"S 171°12'18.7"E	BR	Greymouth	MG132476	
	234	12-Nov-15	Martoni, F & Evans, H	41°45'15.8"S 171°36'57.0"E	NN	Westport	MG132477	
	236	12-Nov-15	Martoni, F & Evans, H	41°45'07.1"S 171°37'14.6"E	NN	Westport	MG132478	
	238	12-Nov-15	Martoni, F & Evans, H	41°14'47.1"S 172°06'43.3"E	NN	Karamea	MG132479	
	244	04-Dec-15	Bulman, SR	45°21'57.5"S 170°50'51.6"E	DN	Moeraki	MG132480	
	429	10-Sep-16	Martoni, F	43°09'23.4"S 172°43'50.5"E	NC	Amberley		

Microscope slide preparation of specimens of both *Psylla frodobagginsi* and *P*. *apicalis* follows the work of Taylor *et al*. [6]. Morphology of adult characters follows the work of Rendón-Mera *et al*. [21]. Photographs were taken using a Nikon DS-Ri2 camera connected to a Nikon SMZ25 microscope. Pictures were the result of stacking images using the software Nikon NIS-Elements D v4.5. The magnification of each picture depended upon the dimensions of the insects and of the morphological character of interest. All the plates were then prepared using the software GIMP version 2.8.14. All psyllid specimens collected, together with the holotype and some of the paratypes, have been deposited in the Lincoln University Entomology Collection (**LUNZ**). Additional paratypes have been deposited at the New Zealand Arthropods Collection (**NZAC**). The holotype of *P*. *apicalis* was provided by the NZAC.

Label data of holotypes and paratypes are reported using the conventions previously stated by Brown [22]: labels are delimited using quotes (‘…’), separate lines are indicated with a solidus (/) while all the metadata are reported in curly brackets ({…}). Reference to COI barcode molecular data gathered prior [3] is reported here for the specific specimens used in the descriptions below, except for the *P*. *apicalis* holotype (Table 1).

### Nomenclatural Acts

The electronic edition of this article conforms to the requirements of the amended International Code of Zoological Nomenclature (ICZN) [23, 24], and hence the new names contained herein are available under that Code from the electronic edition of this article. This published work and the nomenclatural acts it contains have been registered in ZooBank, the online registration system for the ICZN. The ZooBank LSIDs (Life Science Identifiers) can be resolved and the associated information viewed through any standard web browser by appending the LSID to the prefix “http://zoobank.org/”. The LSID for this publication is: urn:lsid:zoobank.org:pub:346EDDD2-AA9C-4C18-8251-36A543EF941E.

The electronic edition of this work was published in a journal with an ISSN and has been archived and is available from the following digital repositories: PubMed Central and LOCKSS.

## Results

From eight of the sampled locations, mostly west and south of the island, morphological examination confirmed that all the psyllids collected belonged to the same ‘large-sized’ morphospecies, later identified as *Psylla apicalis* (Fig 1, black squares) (see below). No other psyllid species was found on these plants. At ten other locations, mostly east and south of the island, all samples collected belonging to the ‘small-sized’ morphospecies were collected and later identified as *Psylla frodobagginsi* (Fig 1, black circles) (see below). Additionally, in three locations on the east and very south of the island (Fig 1, white squares), *Psylla apicalis* and *P*. *frodobagginsi* were collected from the same *S*. *microphylla* plant.

### Taxonomy

#### *Psylla frodobagginsi* Martoni, 2019

**Fig 2:**
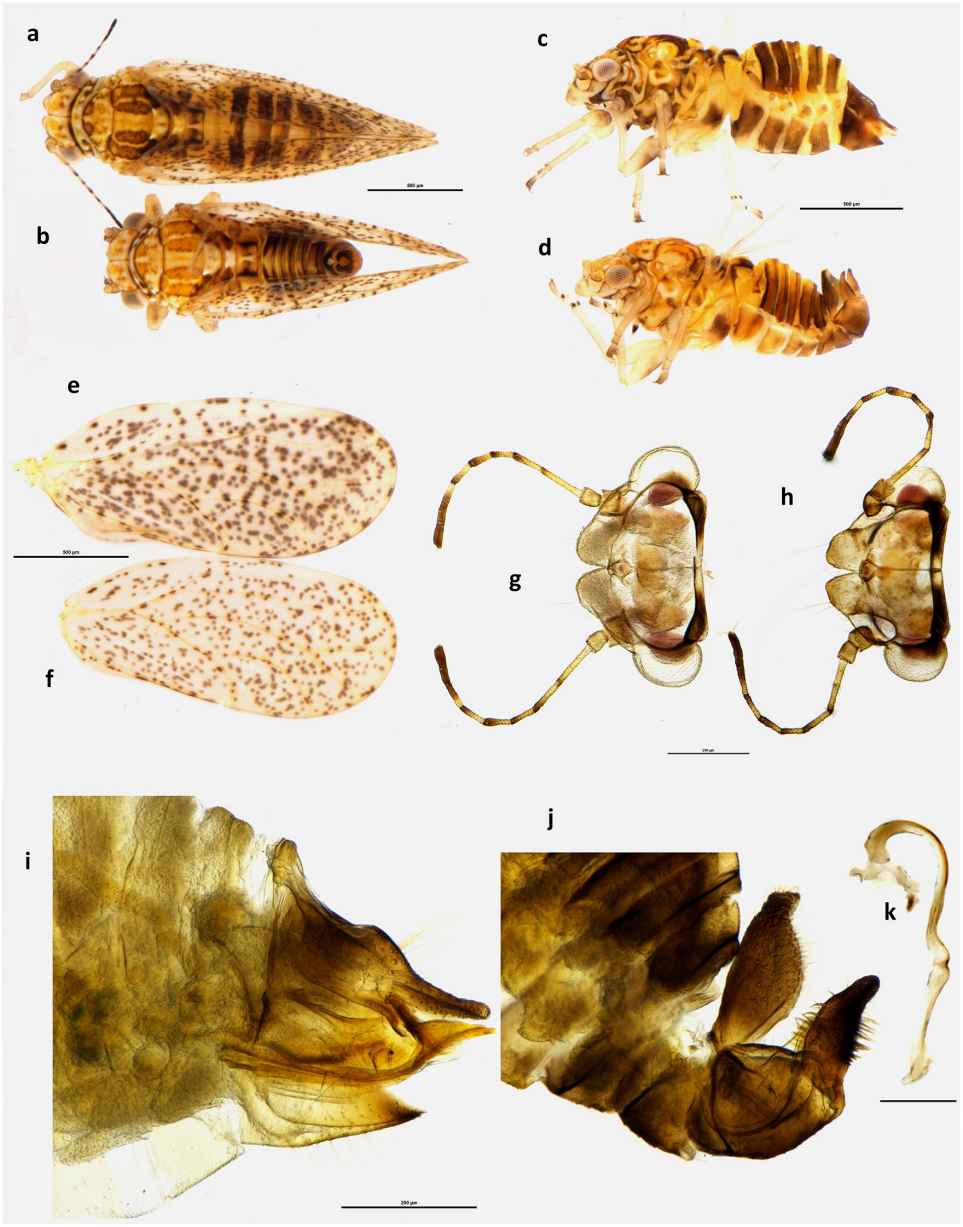
*Psylla frodobagginsi* sp. nov. Habitus dorsal view of female **(a)** and male **(b)**; habitus lateral view of female **(c)** and male **(d)**; wings of female **(e)** and male **(f)**; head dorsal view of female **(g)** and male **(h)**; lateral view of terminalia of female **(i)** and male **(j)**, with particular of the aedeagus **(k)**. Scale bar length = 500μm **(a-f)**, 200μm **(g-j)** and 100μm **(k)**.

urn:lsid:zoobank.org:act:EC986474-A6FF-4484-8E0D-11935DE60B5C (Fig 2).

#### Material examined

*Holotype*: **♂** deposited at the LUNZ. Entire specimen mounted on card triangle. Labels: ‘NEW ZEALAND, DN / Oamaru Botanic Gardens / 45°5’13.47”S 170°58’31.85”E / 2 Nov 2015 H. Evans, F. Martoni / On *Sophora* sp.’ {Printed on white card}. ‘ID 200—Martoni F. 2017 / PhD Thesis. Lincoln University, / Canterbury–New Zealand.’ {Printed on white card}. ‘HOLOTYPE ♂ / *Psylla frodobagginsi* / Martoni 2019’ {Printed on red card}

*Paratypes*: **1 ♀** deposited at the LUNZ. Entire specimen mounted on card triangle. Labels: ‘NEW ZEALAND, DN / Oamaru Botanic Gardens / 45°5’13.47”S 170°58’31.85”E / 2 Nov 2015 H. Evans, F. Martoni / On *Sophora* sp.’ {Printed on white card}. ‘ID 200—Martoni F. 2017 / PhD Thesis. Lincoln University, / Canterbury–New Zealand.’ {Printed on white card}. ‘PARATYPE ♀ / *Psylla frodobagginsi* / Martoni 2019’ {Printed on blue card}; **3 ♂, 3 ♀** deposited at LUNZ. Dissected specimens mounted on microscope slide. Labels: ‘PARATYPE / *Psylla frodobagginsi* / Martoni 2019’ {Hand-written on white card}. ‘NEW ZEALAND, OL Wanaka / Lake front parking. / 10 Nov 2015 –H. Evans, F. Martoni / On *Sophora* sp.’ {Hand-written on white card}; **3 ♂, 3 ♀** deposited at NZAC. Entire specimen mounted on card triangle. Labels: ‘PARATYPE / *Psylla frodobagginsi* / Martoni 2019’ {Printed on white card}. ‘NEW ZEALAND, DN Moeraki / 45°21’57.5”S 170°50’51.6”E / 04 Dec 2015 –S. Bulman / On *Sophora* sp.’ {Printed on white card}.

*Other samples examined for this study*: Countless specimens from the populations listed in Table 1 and Fig 1.

#### Diagnosis

**Fig 3:**
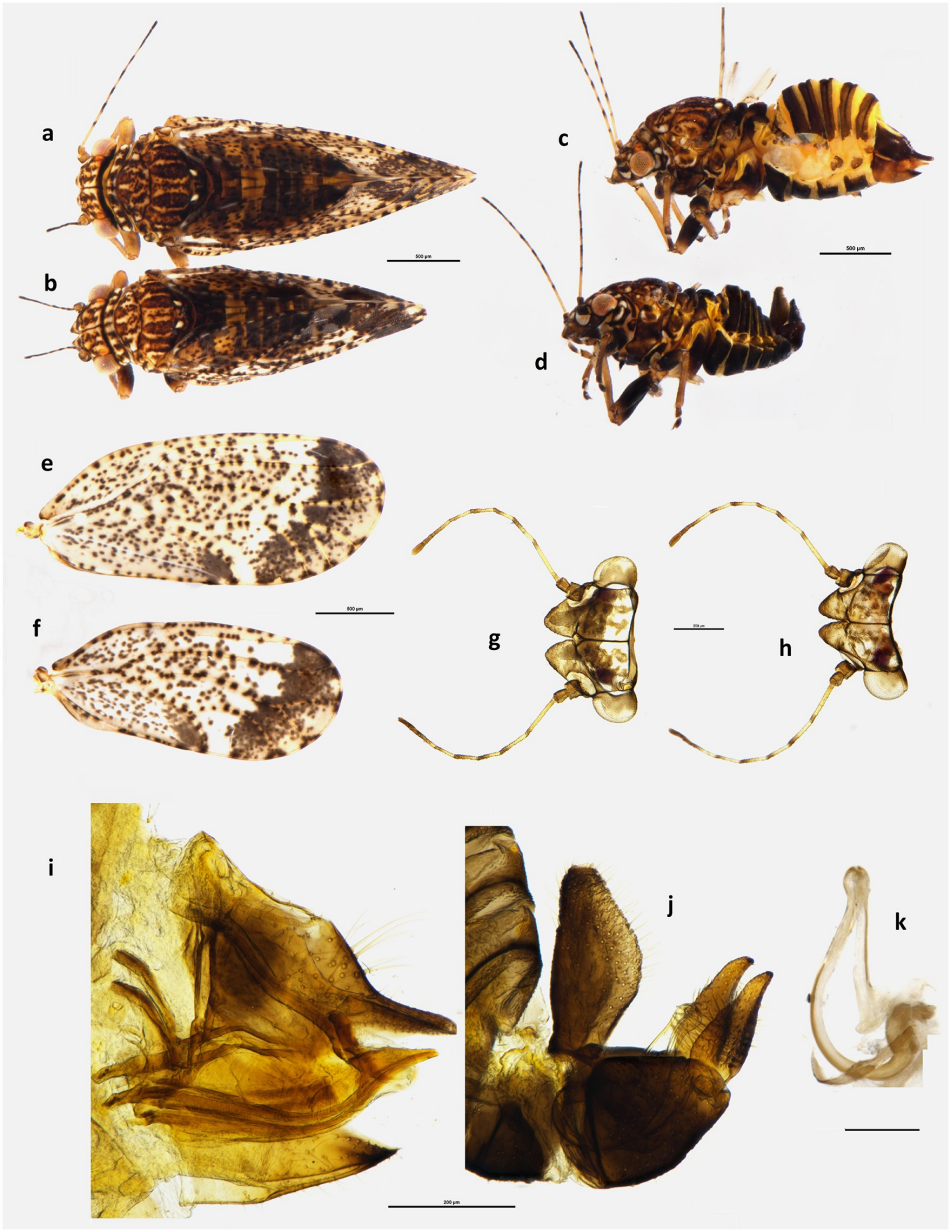
*Psylla* *apicalis*. Habitus dorsal view of female **(a)** and male **(b)**; habitus lateral view of female **(c)** and male **(d)**; wings of female **(e)** and male **(f)**; head dorsal view of female **(g)** and male **(h)**; lateral view of terminalia of female **(i)** and male **(j)**, with particular of the aedeagus **(k)**. Scale bar length = 500μm **(a-f)**, 200μm **(g-j)** and 100μm **(k)**.

*Psylla frodobagginsi* can be identified most easily by its small dimension, light colours, and uniformly spotted wings. *Psylla frodobagginsi* is smaller than *P*. *apicalis*, with males as small as 1.24mm, compared to the 1.65mm of *P*. *apicalis*, and females as small as 1.56mm, against the 2.03mm of *P*. *apicalis*. In addition, *P*. *frodobagginsi* is light coloured (Fig 2a–2d), with orange/light brown bands on pronotum, mesoprescutum, mesoscutum and abdomen, as opposed to dark brown/black bands in *P*. *apicalis* (Fig 3a–3d). Furthermore, *P*. *apicalis* presents a band of merged spots, usually black, crossing the width of the distal part of the wing and a second band, running parallel to the first one (Fig 3e and 3f), where no spots can be seen in the cells, as if the spots present here moved to compose the black band. While the colour of the dark band can vary a lot from black to light brown (as mentioned in [18]), the lack of spots in the area adjacent to the band (Fig 3e and 3f) is a clear diagnostic character that distinguish *P*. *apicalis* from *P*. *frodobagginsi*. Additionally, *P*. *apicalis* has a black spot in the cell cu1 (Fig 3e and 3f). *Psylla frodobagginsi*, on the contrary, presents the forewing uniformly covered in spots, without any band or darker region (Fig 2e and 2f).

Diagnosis is also supported by *P*. *frodobagginsi* presenting a number of characters that have a rounded shape, such as the tip of the female proctiger, the tip of the male parameres and the genal processes. In *P*. *apicalis* these are conical and pointy (Fig 3g and 3h) while in *P*. *frodobagginsi* these are rounded and more irregular, especially in the males (Fig 2g and 2h). The female proctiger of *Psylla frodobagginsi* ends in a rounded tip (Fig 2i), while that of *P*. *apicalis* appears to be slightly pointy (Fig 3i). Similarly, the apical part of the male parameres that are more rounded in *P*. *frodobagginsi* (Fig 2j and 2k), while they are pointy in *P*. *apicalis* (Fig 3j and 3k).

#### Colouration

Body light coloured, mostly yellowish/orange. Head yellow and pronotum of similar colour except for four darker spots (two at each extremity). Antennae yellow with apical part of segments 1–6 darker and segments 7 and 8 completely dark brown/black. Genal processes yellow and eyes just slightly darker. Mesothoracic prescutum of the same colour except for two triangular darker spots (orange/brown) in frontal position. Mesothoracic scutum yellowish orange with four dark bands running parallel to the head/terminalia axis. Abdomen yellowish/orange with darker bands on each segment. Wings are hyaline uniformly spotted. Both male and female terminalia are orange/brown.

#### Structure

Body very small, with females well under 2mm and males under 1.5mm. Head: vertex generally twice as wide as long. Genal processes short and quite rounded, more so in males than females. Antennae moderately long, more so in males (up to 1.3 times the width of the head) than in females (maximum 1.1 times). Thorax short and broad. Fore wing at least three times as long as width of head and between 2.2 and 2.4 times as long as wide. Veins Rs longer than M, and M1+2 on average half as long as vein M, but quite variable.

Male terminalia quite slender, with proctiger longer than parameres. Parameres thin and slender internally covered in setae that, in lateral view, can be seen protruding frontally and caudally. The tip of the parameres is rounded and pointing caudally. Female terminalia are straight and moderately long with proctiger longer than the subgenital plate (up to 1.36 times), wider in the proximal part and narrow to the point with a rounded bump separating the circum-anal ring from the narrower tip of the proctiger.

#### Measurements

Measurements are in mm (5 ♂, 5 ♀). Length of body (vertex to terminalia) ♂ 1.24–1.41, ♀ 1.56–1.72; length of body (vertex to apex of folded wings) ♂ 1.67–1.88, ♀ 1.98–2.11; width of head (HW) ♂ 0.52–0.54, ♀ 0.56–0.60; length of genal processes (GCL) ♂ 0.09–0.10, ♀ 0.10–0.11; length of vertex (VL) ♂ 0.16–0.18, ♀ 0.18–0.20; width of vertex (VW) ♂ 0.32–0.35, ♀ 0.36–0.41; length of antenna (AL) ♂ 0.64–0.72, ♀ 0.61–0.65; length of fore wing ♂ 1.62–1.66, ♀ 1.79–1.88; width of fore wing ♂ 0.69–0.74, ♀ 0.77–0.83; length of vein Rs ♂ 0.86–1.00, ♀ 1.05–1.09; length of vein M (M) ♂ 0.66–0.82, ♀ 0.64–0.88; length of vein M1+2 (M1) ♂ 0.34–0.39, ♀ 0.32–0.52; marginal width of cell m1 ♂ 0.18–0.24, ♀ 0.20–0.24; marginal width of cell cu1 ♂ 0.36–0.43, ♀ 0.40–0.44; length of vein Cu1b ♂ 0.20–0.26, ♀ 0.24–0.28; length (height) of proctiger (PL) ♂ 0.24–0.28; length of paramere ♂ 0.17–0.18; length of proximal aedeagal segment ♂ 0.30–0.34; length of distal aedeagal segment ♂ 0.11–0.16; length of proctiger (PL) ♀ 0.32–0.34; length of circumanal ring (CL) ♀ 0.16–0.17; length of subgenital plate (SL) ♀ 0.24–0.28.

#### Ratios

GCL:VL ♂ 0.50–0.59, ♀ 0.53–0.61; VL:VW ♂ 0.47–0.56, ♀ 0.47–0.53; VL:HW ♂ 0.30–0.35, ♀ 0.31–0.34; AL:HW ♂ 1.23–1.33, ♀ 1.05–1.12; PL:HW ♂ 0.46–0.52, ♀ 0.55–0.61; PL:CL ♀ 1.94–2.12; PL:SL ♀ 1.21–1.36; WL:HW ♂ 3.00–3.17, ♀ 3.08–3.24; WL:WW ♂ 2.24–2.39, ♀ 2.19–2.32; Rs:M ♂ 1.20–1.36, ♀ 1.20–1.70; M1:M ♂ 0.41–0.59, ♀ 0.36–0.81.

#### Etymology

The name *Psylla frodobagginsi* refers to Frodo Baggins, one of the main characters of J. R. R. Tolkien’s literary trilogy commonly referred to as “The Lord of the Rings” [25, 26, 27]. In the books, Frodo Baggins is a hobbit, a member of a fictitious race similar to humans, but of smaller size. The name was chosen due to the smaller size of this psyllid species, together with the fact it is native to New Zealand. The cinematographic sets for P. Jackson’s movie trilogy “The Lord of the Rings” were for the majority placed in the South Island, reflecting the proportional distribution of the psyllid’s endemic host *Sophora microphylla*.

#### Distribution

*Psylla frodobagginsi* was recorded in the South Island of New Zealand from a total of 13 locations (Fig 1, Table 1). From three of these, this species was present at the same time on the same individual *Sophora microphylla* plant as *P*. *apicalis*. No collections were performed in the North Island. However, due to the presence of kōwhai there, *P*. *frodobagginsi* is hypothesised to be present, as well.

#### Host plant

All the populations examined in this study were collected from the plant *Sophora microphylla*, the small-leaved kōwhai, a plant endemic to New Zealand. Nonetheless, with previous records of *P*. *apicalis* collected also from *S*. *tetraptera* [17, 18] and *S*. *prostrata* Buchanan [17], these plants are to be considered possible host plants, too. However, probably due to the time of the year when collections were performed, no immature life stages were found. Such a lack of nymphs would suggest caution in the host plant assessment. Generally, in fact, a psyllid host plant is defined as ‘the plant on which psyllids can complete their life cycle, from nymphs to adults’ [28]. Nonetheless, considering the high number of adult individuals collected on the same plant species at a wide range of locations, and the absence of *Psylla frodobagginsi* on other plants previously examined [3], *Sophora microphylla* is hypothesised here as the host plant of *P*. *frodobagginsi*.

#### *Psylla apicalis* (Ferris and Klyver, 1932)

(Figs 3 and 4).

**Fig 4:**
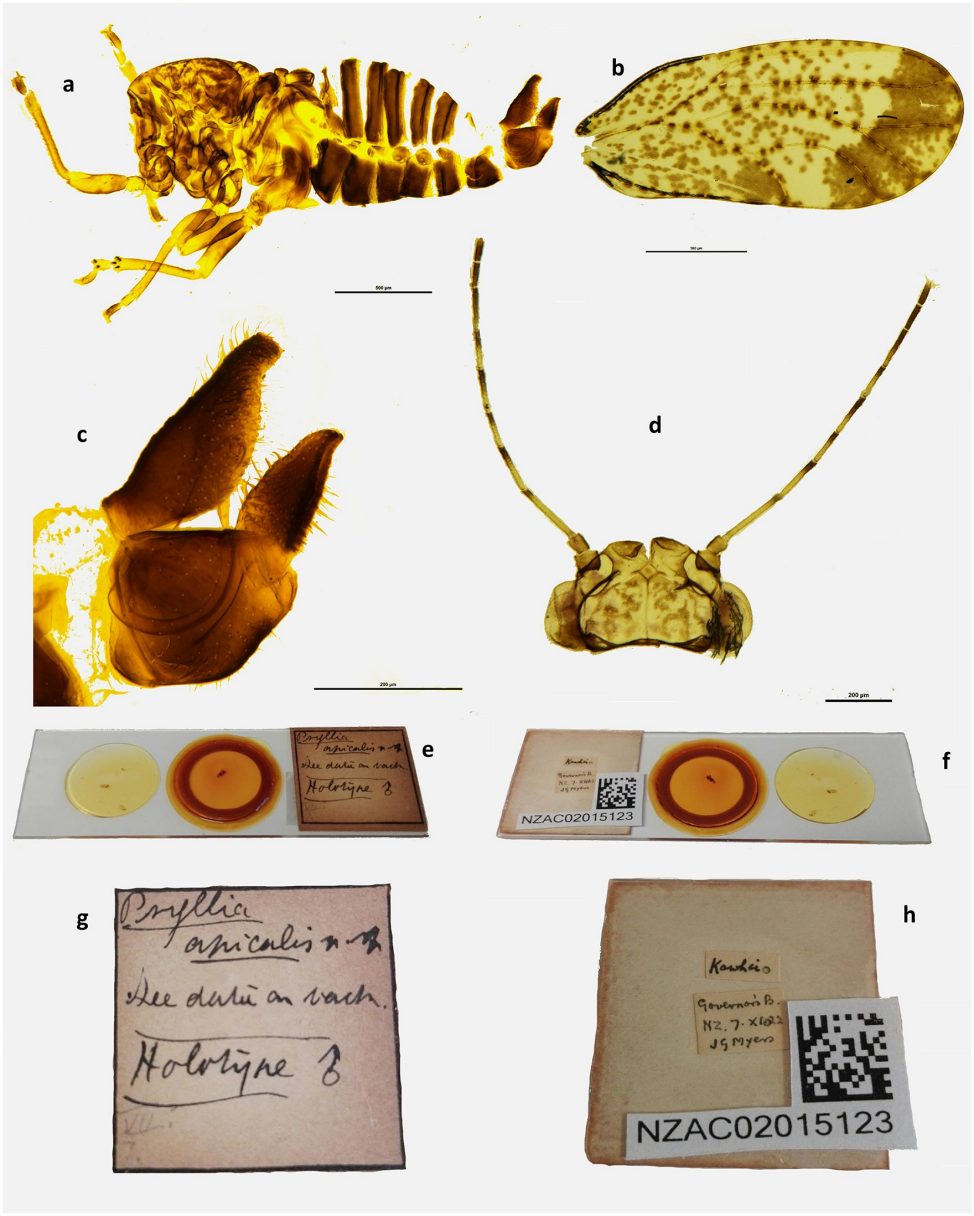
*Psylla apicalis* holotype (male). Habitus lateral view **(a)**; wing **(b)**; terminalia lateral view **(c)**; head dorsal view **(d)**; slide front **(e)** and back **(f)** with frontal **(g)** and posterior **(h)** labels. Scale bar length = 500μm **(a-b)** and 200μm **(c-d)**.

#### Material examined

*Holotype*: **♂** preserved at the NZAC. Dissected specimen mounted on microscope slide. Labels: ‘Psyllia (sic)/ apicalis n. sp. / see data on back / holotype **♂’** {hand written on white card, on front}. ‘Kōwhai / Governors’ B. / NZ. 7.X.1922 / JG Myers’ {hand written on white card, on back}. ‘NZAC02015123’ {typed on white card, on back} (Fig 4).

*Samples measured for this study*: **5 ♂, 5 ♀** deposited at LUNZ. Dissected specimens mounted on microscope slide. Labels: ‘NEW ZEALAND, MC, Christchurch / Canterbury University campus / 01 September 2015 / on Kōwhai, SDJ Brown’ {printed on white card}. (Fig 3).

*Other samples examined for this study*: Countless specimens from the populations listed in Table 1 and Fig 1.

### Updated description

#### Colouration

Body dark orange/black, with head and thorax showing black striped on a dark orange background. Head orange uniformly covered in darker spots. Eyes dark orange/brown. Genal processes brown to black. Pronotum darkened by the presence of seven darker spots (three at each extremity and one in the centre). Antennae yellow with apical part of segments 1–6 darker and segments 7 and 8 completely dark brown/black. Mesothoracic prescutum presenting the same two triangular darker spots of *P*. *frodobagginsi* with the addition of a multitude of black spots (Fig 3a and 3b). Mesothoracic scutum orange/brown with four black bands running parallel to the head/terminalia axis and a thin black line in the middle. Abdomen yellowish/orange with black wide bands on each segment that almost completely cover the yellow colour. Wings are hyaline, with dark brown-black spots that are not uniformly distributed. Spots merge to form a black diagonal band crossing the wing from cell cu1 to r1 and a black spot in the cu2 cell. The colour of band and spot can be lighter at times, almost resembling the colouration of *P*. *frodobagginsi*, but in *P*. *apicalis* there is an additional contiguous band, parallel to the dark one, that shows an absence of spots except on the veins. This clearer band on the wing is not hyaline, but completely transparent. Both male and female terminalia are dark brown to black.

#### Structure

Body larger than *P*. *frodobagginsi*, with males of this species generally bigger than females of *P*. *frodobagginsi*. All measurements were generally greater than those of *P*. *frodobagginsi*, some with no overlap of the ranges and showing a clear gap; in particular the length of the body for both sexes. On the other hand, ratios appear similar to those of *P*. *frodobagginsi* except for the Rs:M in males where that for *P*. *frodobagginsi* were between 1.20mm–1.36mm while those of *P*. *apicalis* are between 1.43mm–1.53mm.

#### Measurements

Measurements are in mm (5 ♂, 5 ♀) and the measurements of the Holotype (♂, Fig 4) are reported in brackets in bold, when it was possible to make the measurement. Length of body (vertex to terminalia) ♂ 1.65–1.90, ♀ 2.03–2.24; length of body (vertex to apex of folded wings) ♂ 2.15–2.43, ♀ 2.59–2.74; width of head (HW) ♂ 0.60–0.67 **(0.63)**, ♀ 0.66–0.71; length of genal processes (GCL) ♂ 0.12 **(0.1)**, ♀ 0.13–0.14; length of vertex (VL) ♂ 0.17–0.21 **(0.2)**, ♀ 0.22–0.23; width of vertex (VW) ♂ 0.38–0.40 **(0.41)**, ♀ 0.40–0.51; length of antenna (AL) ♂ 0.93–1.02 **(1.11)**, ♀ 0.89–0.91; length of fore wing ♂ 1.88–1.98 **(1.92)**, ♀ 2.09–2.40; width of fore wing ♂ 0.79–0.86 **(0.84)**, ♀ 0.92–1.05; length of vein Rs ♂ 1.10–1.23 **(1.20)**, ♀ 1.28–1.39; length of vein M (M) ♂ 0.75–0.86 **(0.83)**, ♀ 0.87–0.96; length of vein M1+2 (M1) ♂ 0.42–0.51 **(0.47)**, ♀ 0.49–0.62; marginal width of cell m1 ♂ 0.23–0.27 **(0.20)**, ♀ 0.24–0.32; marginal width of cell cu1 ♂ 0.40–0.51 **(0.48)**, ♀ 0.50–0.55; length of vein Cu1b ♂ 0.24–0.28 **(0.28)**, ♀ 0.30–0.37; length (height) of proctiger (PL) ♂ 0.31–0.33 **(0.33)**; length of paramere ♂ 0.20–0.22 **(0.22)**; length of proximal aedeagal segment ♂ 0.41–0.45 **(0.44)**; length of distal aedeagal segment ♂ 0.18–0.21 **(0.21)**; length of proctiger (PL) ♀ 0.38–0.42; length of circumanal ring (CL) ♀ 0.17–0.21; length of subgenital plate (SL) ♀ 0.30–0.35.

#### Ratios

GCL:VL ♂ 0.57–0.71 **(0.50)**, ♀ 0.56–0.61; VL:VW ♂ 0.45–0.52 **(0.51)**, ♀ 0.45–0.57; VL:HW ♂ 0.27–0.32 **(0.33)**, ♀ 0.32–0.34; AL:HW ♂ 1.50–1.58 **(1.33)**, ♀ 1.32–1.35; PL:HW ♂ 0.46–0.53 **(0.48)**, ♀ 0.57–0.59; PL:CL ♀ 1.90–2.28; PL:SL ♀ 1.14–1.27; WL:HW ♂ 2.92–3.27 **(3.05)**, ♀ 3.12–3.64; WL:WW ♂ 2.26–2.48 **(2.29)**, ♀ 2.25–2.35; Rs:M ♂ 1.43–1.53 **(1.45)**, ♀ 1.39–1.53; M1:M ♂ 0.49–0.65 **(0.57)**, ♀ 0.53–0.66.

## Discussion

The results presented here enable the morphology of *Psylla frodobagginsi* to be distinguished from that of *P*. *apicalis*. This is in agreement with recently presented molecular data [3] where a COI divergence of 7–8% suggested the presence of two separate taxa on *Sophora*. Furthermore, this work finally allows the doubts presented by Ferris and Klyver [18], when first describing *P*. *apicalis* 87 years ago, to be resolved and confirms that there are two psyllid species on kōwhai. With *Psylla frodobagginsi*, there are now 74 described psyllid species present in New Zealand of which 41 are endemic.

In a taxonomical context, the description of *P*. *frodobagginsi* highlights the need of a review of the genus *Psylla* in New Zealand. Firstly, because the number of species is suspected to be higher than the three species described so far, possibly including a number of undescribed taxa found associated with *Carmichaelia* spp., another group of the Fabaceae native to New Zealand (as previously suggested [3]). Secondly, based on divergent morphological and molecular characters, these species probably do not belong to the European genus *Psylla* at all, and they will need to be moved under a new New Zealand endemic genus (as previously suggested [8]). This is consistent with early quandaries about attribution of the two New Zealand described species to the *Psylla*. While Tuthill, who described *P*. *carmichaeliae* in 1952 (Tuthill 1952) found the species congeneric to *P*. *apicalis* he also highlighted similarities between both New Zealand species and *Euphalerus nidifex* Schwarz [29]. Nonetheless, he did not consider this sufficient to move the New Zealand species to *Euphalerus* [29]. Later, in 1985, Dale re-examined *P*. *apicalis* and *P*. *carmichaeliae* and moved them to the existing genus *Euphalerus* (Macrocorsinae) [17]. However, *Euphalerus* was subsequently redefined to include only species from the New World [30], leaving the New Zealand species in a genus requiring revision. Thus, the original doubt of Tuthill [29] was confirmed, that the Australasian species considered *Euphalerus* were not congeneric with the type species *E*. *nidifex* from Central America. Tuthill’s uncertainty was mostly due to morphological variation in the last instar of the immature life stages of the two New Zealand species, *P*. *apicalis* and *P*. *carmichaeliae*. However, characters such as the eight-segmented antennae and the marginal setae on the caudal plate, would eventually contribute to placing these taxa in the subfamily Psyllinae, but outside *Psylla* [8]. This, together with the new information in this current description, therefore supports the accumulation of evidence that a broader, world-wide study is necessary to elucidate the taxonomy of the family Psyllidae, comprising *Arytainilla*, *Heteropsylla*, *Baeopelma*, *Psylla* sensu stricto, and the New Zealand ‘*Psylla’*.

The South Island-wide geographic distribution of the specimens collected, together with the presence of both *Psylla apicalis* and *P*. *frodobagginsi* on the same individual plant in multiple locations, suggests that both species are occupying the same ecological niche. It remains quite unusual for the same plant to host multiple psyllid species [31]). However, in New Zealand this has been observed for other species, such as the adventives *Psyllopsis fraxini* and *P*. *fraxinicola*, both hosted by *Fraxinus excelsior* [8]. On the other hand, polyphagy appear to be a more common trait in psyllid pest species such as *Bactericera cockerelli*, the tomato potato psyllid (TPP), and *Diaphorina citri*, the Asian citrus psyllid [1]. The overlapping distribution of the two species associated with kōwhai, together with the small size of psyllids making it difficult to appreciate their distinctiveness outside of dedicated taxonomic studies such as this, might be the main reason why they have been considered the same species until today. The misconception has likely been perpetuated by the common name “kōwhai psyllid” which has been used indiscriminately to refer to any psyllid found on *S*. *microphylla*. Without ongoing, targeted taxonomic studies, species such as *P*. *frodobagginsi* effectively ‘hide in plain sight’ contrary to a common notion that new species are only to be found in unchartered or non-urban environments. Here we propose to refer to *P*. *frodobagginsi* as the “hobbit kōwhai psyllid”, as compared to *P*. *apicalis* that could become the “greater kōwhai psyllid”. Finally, the focus of this study on South Island was largely a consequence of *S*. *microphylla*, which is the most common of the eight *Sophora* species, in the wild being largely restricted to the South Island. However, as New Zealand’s national plant it is a common ornamental throughout the country. Therefore, additional sampling to include the North Island would provide some interesting information not only about the true distribution of both of these kōwhai-hosted psyllids in New Zealand, but also about how well, or consistently, despite a large range in climatic conditions they share the same ecological niche.
